# Assessment of Natural Language Processing Methods for Ascertaining the Expanded Disability Status Scale Score From the Electronic Health Records of Patients With Multiple Sclerosis: Algorithm Development and Validation Study

**DOI:** 10.2196/25157

**Published:** 2022-01-12

**Authors:** Zhen Yang, Chloé Pou-Prom, Ashley Jones, Michaelia Banning, David Dai, Muhammad Mamdani, Jiwon Oh, Tony Antoniou

**Affiliations:** 1 Data Science and Advanced Analytics Unity Health Toronto Toronto, ON Canada; 2 Division of Neurology Department of Medicine St. Michael's Hospital Toronto, ON Canada; 3 Li Ka Shing Knowledge Institute Unity Health Toronto Toronto, ON Canada; 4 Faculty of Medicine University of Toronto Toronto, ON Canada; 5 Leslie Dan Faculty of Pharmacy University of Toronto Toronto, ON Canada; 6 Institute of Health Policy, Management, and Evaluation University of Toronto Toronto, ON Canada; 7 Department of Neurology Johns Hopkins University Baltimore, MD United States; 8 Department of Family and Community Medicine Unity Health Toronto Toronto, ON Canada; 9 Department of Family and Community Medicine University of Toronto Toronto, ON Canada

**Keywords:** machine learning, multiple sclerosis, natural language processing

## Abstract

**Background:**

The Expanded Disability Status Scale (EDSS) score is a widely used measure to monitor disability progression in people with multiple sclerosis (MS). However, extracting and deriving the EDSS score from unstructured electronic health records can be time-consuming.

**Objective:**

We aimed to compare rule-based and deep learning natural language processing algorithms for detecting and predicting the total EDSS score and EDSS functional system subscores from the electronic health records of patients with MS.

**Methods:**

We studied 17,452 electronic health records of 4906 MS patients followed at one of Canada’s largest MS clinics between June 2015 and July 2019. We randomly divided the records into training (80%) and test (20%) data sets, and compared the performance characteristics of 3 natural language processing models. First, we applied a rule-based approach, extracting the EDSS score from sentences containing the keyword “EDSS.” Next, we trained a convolutional neural network (CNN) model to predict the 19 half-step increments of the EDSS score. Finally, we used a combined rule-based–CNN model. For each approach, we determined the accuracy, precision, recall, and F-score compared with the reference standard, which was manually labeled EDSS scores in the clinic database.

**Results:**

Overall, the combined keyword-CNN model demonstrated the best performance, with accuracy, precision, recall, and an F-score of 0.90, 0.83, 0.83, and 0.83 respectively. Respective figures for the rule-based and CNN models individually were 0.57, 0.91, 0.65, and 0.70, and 0.86, 0.70, 0.70, and 0.70. Because of missing data, the model performance for EDSS subscores was lower than that for the total EDSS score. Performance improved when considering notes with known values of the EDSS subscores.

**Conclusions:**

A combined keyword-CNN natural language processing model can extract and accurately predict EDSS scores from patient records. This approach can be automated for efficient information extraction in clinical and research settings.

## Introduction

Multiple sclerosis (MS) is the most common cause of neurological disability in young adults in the developed world [1]. Although the majority of individuals present initially with relapsing-remitting disease, neurological disability can accumulate over time, resulting in significant functional impairment in a substantial portion of people with MS [1,2]. However, there is considerable individual heterogeneity in MS disease progression, such that validated measures of disability are required to monitor functional decline and response to disease-modifying therapies.

The Kurtzke Expanded Disability Status Scale (EDSS) is the most widely used validated measure to quantify and monitor changes in MS-related disability over time [3,4]. The EDSS is a clinician-administered ordinal rating system that quantifies disability in 8 functional systems, increasing from 0 (no disability) to 10 (death due to MS) in increments of 0.5 units. EDSS subscores can also be determined for each of the individual functional systems comprising the total score, using a scale that ranges from 0 to 5 or 6 [3,4]. Because the EDSS score is used for both clinical and research purposes, it is typically extracted or derived manually from electronic medical records and transcribed in clinical and research databases to monitor trends in disease evolution and response to treatment [5-7]. However, the EDSS score may not be determined at all visits, introducing missing data when patient records are used for research and clinical monitoring [8]. Moreover, extracting and deriving the EDSS score from patient records is time-consuming and inefficient because of the unstructured nature of clinical records [9].

Natural language processing is a field of artificial intelligence that is increasingly being applied to extract and transform unstructured notes in electronic medical records into coded data that can be used for clinical, quality improvement, and research purposes [10,11]. Natural language processing has been studied in a variety of clinical settings, including oncology, emergency medicine, and primary care, for applications as varied as case ascertainment, risk assessment, and disease staging [12-16]. Within the field of MS, comparatively few studies have investigated the use and performance of natural language models. Specific areas of application have included identifying patients with MS from clinical databases, extracting disease-specific variables, detecting genotype-phenotype associations for MS from an electronic medical record–linked DNA biorepository, identification and sentiment analysis of MS-related content on social media, biomedical literature mining, and using clinical variables to derive a disease severity score [9,17-24]. Existing studies thus far have largely evaluated rule-based natural language processing approaches, wherein clinicians provide keywords and a predetermined set of rules to locate specific text in a note that denotes a particular finding as either present or absent. Deep learning natural language processing approaches, in which machine learning algorithms are trained to capture specific outcomes from text, have been less well studied in the MS field. Our objective was to compare rule-based and deep learning natural language processing algorithms for detecting and predicting the total EDSS score and EDSS functional system subscores from clinic notes.

## Methods

### Setting and Data Sources

The Barlo MS Centre of Unity Health Toronto is one of the largest MS clinics in Canada, providing specialized care to over 7000 Ontario residents living with MS. The clinic database contains comprehensive information on all patients, including demographic data, relapse and treatment history, imaging results, and findings from neurological examinations, including EDSS and functional system scores. For this study, we extracted all clinical notes generated for patients seen at the clinic between June 2015 and July 2019, and randomly divided all notes in the study period into training (80%) and test (20%) data sets. We divided notes at the patient level to prevent data leakage (ie, same patient appearing in both training and test data sets).

### Data Preprocessing

To prepare notes for rule-based and deep learning natural language processing, we first removed all redundant information, including patient and physician names within the header and footer of each note, date and time of visit, fax number, and document number. We also removed identifying information such as home addresses, phone numbers, patient identification number, and dates of birth and electronic signatures, as well as nonletter characters such as punctuation, symbols, and left-over whitespace. Next, we removed stop words using the Natural Language Toolkit default list [25]. Stop words are commonly used terms (eg, “and,” “it,” “the,” etc) that have little value with respect to the meaning of clinical text. We completed these steps so that only the most relevant parts of the document would be provided as input to the text classification model. Finally, we encoded each note into a sequence of integers, setting the maximum sequence length to 1000 words, which is within the limit of most notes included for study. We zero-padded sequences with smaller word counts, and removed the last few words when the sequence count exceeded the maximum length. Preprocessing steps were automated, applicable to the test-time/application-time, and did not require manual review.

### Natural Language Processing

We compared the performance characteristics of 3 natural language processing models in outputting 1 EDSS score for each note. First, we used a rule-based approach, wherein the preprocessed text was divided into sentences, and extracted the EDSS score on the first occasion when “EDSS” and a numeric value between 0.0 and 10.0 appeared in the same sentence. To extract EDSS functional system subscores, MS clinic staff were consulted to develop rules that paired keyword patterns representing clinical findings relevant to a specific functional system (eg “ataxia” for the cerebellar subscore and “indwelling catheter” for the bowel and bladder score) with adjectives denoting the varying levels of disability related to each functional system, such as “mild,” “moderate,” or “significant.” These rules were based on Neurostatus definitions and scoring for neurological examinations [26]. Using this approach, EDSS subscores were extracted or derived for each functional system.

**Figure 1:**
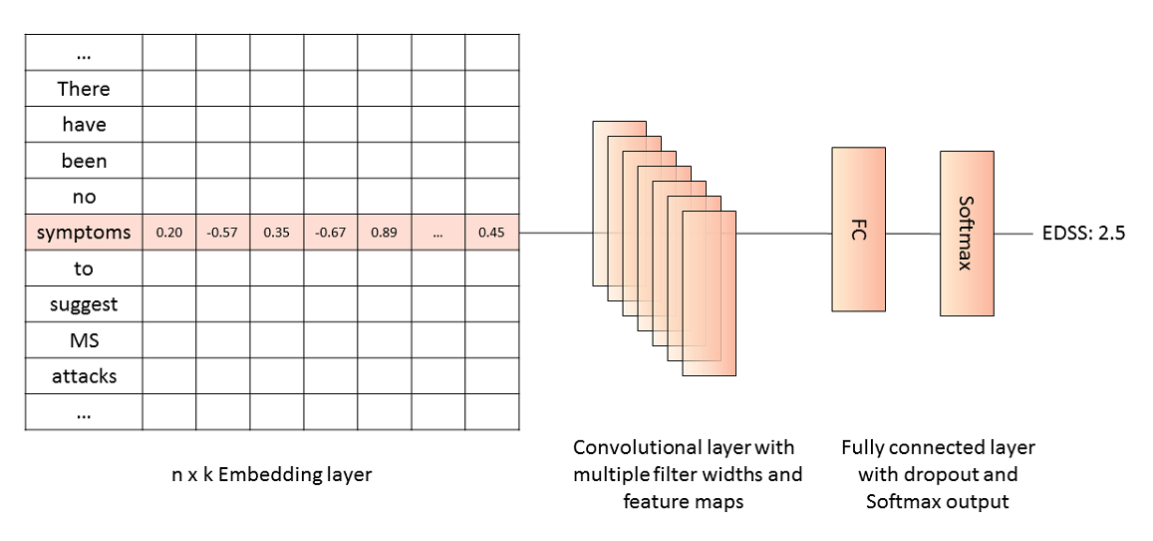
Convolutional neural network model structure. EDSS: Expanded Disability Status Scale.

Because it is possible that multiple keywords can appear in the same note (eg, “EDSS was 5.0 in the previous visit. …EDSS is 6.0 in this visit.”), the rule-based approach may result in errors when extracting the most recent EDSS score, highlighting the potential limitations of this approach and the need to evaluate alternative models. We therefore trained separate convolutional neural network (CNN) models to predict the 19 half-step increments of the total EDSS score and the functional system subscores. CNNs are artificial neural networks that are being increasingly used for applications as varied as image detection and natural language processing [27-29]. In the case of the latter, text must first be converted into a numerical form known as a word vector before it can be fed into a CNN model. To do this, we experimented with various approaches, including Bidirectional Encoder Representations from Transformers (BERT) [30], BioBERT [31], deep contextualized word representations (Embeddings from Language Models [ELMo]) [32], and pretrained Word2Vec (trained on PubMed, Wiki, and PubMed Central) [33]. A comparison of these approaches found that Word2Vec trained on our hospital data had superior performance and runtime relative to the other approaches. Moreover, Word2Vec embeddings trained on our data were able to capture semantic relationships between MS-related terms. For example, the terms RRMS (“relapsing-remitting multiple sclerosis”), AMS (“active multiple sclerosis”), and CIS (“clinically isolated syndrome”) are identified as nearest neighbors of the term “MS,” using our approach. We therefore trained a 200-dimensional Word2Vec embedding with all neurologist specialty notes from the clinic using Gensim [34]. Word2Vec is a 2-layer neural network net that transforms inputted text into numerical vectors, or embeddings, of a given size (eg, 200 dimensions) that can be processed by CNNs [35]. This is done by grouping the vectors mathematically based on word similarity, with similar words being closer to each other when mapped in multidimensional space, while unrelated words are separated by greater distance. For all of the CNN models, we used 200-dimensional Word2Vec embeddings trained on all clinical notes from the MS clinic. Word embeddings were trained using a window size of 10 and a minimum count of 2, yielding an embedding matrix with a dimension of 1000×200, reflecting the maximum sequence length of 1000 words, that acted as an embedding layer in the CNN models. We chose a 1000-word maximum sequence length based on premodeling determinations of the word count of the consult notes comprising our data set demonstrating that most notes fell within this limit. The CNN model is based on a well-known CNN structure used for sentence classification (Figure 1) [29]. First, a section of the note is represented as a numeric feature (ie, word embedding with a dimension of 1000×200). Next, convolutional layers with multiple filters of different kernel sizes (sizes 3, 4, and 5) are applied to obtain multiple features (with dropout rate 0.5 and maximum pooling on each of the convolutions). Features are then passed to a fully connected layer whose output is the probability distribution over the list of EDSS classes. Therefore, in addition to the embedding layer, CNN models also contained convolutional layers with maximal pooling and fully connected layers with Softmax output (Figure 1) [29]. We implemented the model using Keras 2.0 API [36], and trained the model using the RMSprop optimizer and early stopping to prevent overfitting from too many iterations. We experimented with different learning rates, epochs, batch sizes, and patience for early stopping, choosing the hyperparameters that delivered the best accuracy for our test data. We also tried shallow neural networks (unigram features and a cutoff of 5000 features ordered by term frequency) with term-frequency inverse document frequency features and recurrent neural networks (RNNs) for our study. In the case of the former, we found that these models did not adequately represent word relations and context-based information. Moreover, these approaches created extremely high dimensional sparse input vectors. Although findings with RNNs were comparable, we elected to proceed with the CNN and Word2Vec approach because these models were faster to train.

**Figure 2:**
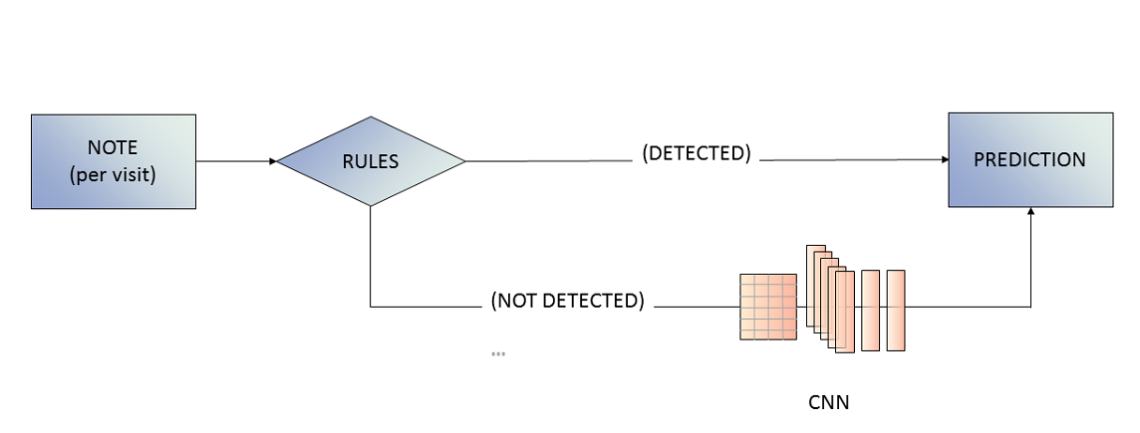
Combined rule-based–CNN model. CNN: convolutional neural network.

Finally, we used a keyword-CNN model to ascertain whether the combination of the 2 approaches would yield better performance metrics than either model alone. We reasoned that a combined model would balance the strengths and limitations of each model separately. Specifically, while CNN models perform well with large data volumes and are less time-intensive than rule-based approaches, these models typically lack transparency and explainability, leaving users with little understanding of how predictions and decisions are made. Moreover, CNN models may not perform well when data volumes are small, such as for patients at the highest extremes of EDSS scores. In contrast, while rule-based approaches are transparent and explainable (ie, extracted keyword patterns in notes can be shown to users), and have good performance for rare outcomes, they will predict mostly unknown results when keywords are not explicitly found in the reference text. To account for these strengths and weaknesses, we developed a combined model that involves 2 steps. First, the model uses a rule-based approach to detect whether the EDSS score is explicitly written in a given note. In such a case, the model outputs the extracted EDSS score. In the event that keywords are not explicitly written, the note is passed on to the CNN, which will provide a prediction for the EDSS score (Figure 2).

### Statistical Analysis

After training, all models were evaluated on the 3493 notes comprising the test set. Our primary outcome was the performance of each model for abstracting and/or deriving the total EDSS score. We determined the accuracy, precision, recall, and F-score of each model compared with the reference standard, which was the manually labeled EDSS scores in the clinic database. Accuracy is the ratio of correct predictions made (ie, true positives plus true negatives) to the total number of predictions made (ie, sum of true positives, false positives, true negatives, and false negatives). For total EDSS scores, predictions were considered accurate if they were identical to those recorded in patient records. For functional subscores, predictions were considered accurate if they were within +/−1 of their referent values. Precision is calculated by dividing the number of true positive predictions by the sum of true and false positives, whereas recall is defined as the number of true positives over the total number of positives (ie, sum of true positives and false negatives). To determine precision and recall, we considered each score as a class, and obtained true positive, false positive, true negative, and false negative rates for each class. Finally, the F-score is a metric that combines precision and recall into a single number using the harmonic mean, thereby taking both false positives and false negatives into account. Compared with accuracy, the F1-score provides a more robust measure of incorrectly classified cases in imbalanced class settings such as ours. In all cases, we determined macro average performance measures, obtained by first calculating each class metric and then taking the average of these. We used Pitman permutation tests to determine whether model differences in accuracy and F1-scores were statistically significant [37]. In secondary analyses, we determined the performance of each model in abstracting functional system EDSS subscores. In a sensitivity analysis, we replicated our analyses using 10-fold cross-validation on the training set. For each fold, we used 90% of the notes for training and 10% for validation, and then applied the hyperparameters producing the best results in the cross-validation toward evaluating the test set.

### Ethics Approval

This study was approved by the Research Ethics Board of Unity Health Toronto, Toronto, Canada (reference #16-371).

## Results

Our data set comprised 17,452 clinic notes for 4906 patients seen at the MS clinic between June 2015 and July 2019. Overall, the mean age of the patients was 49.5 (SD 12.4) years, and 3534 (72%) were female. The majority of notes (n=10,881, 62.3%) had an EDSS score explicitly dictated. There was considerable class imbalance in the EDSS labels, with 13,880 (79.5%) and 1386 (7.9%) scores being in the range of 0.0 to 4.0 and above 6.0, respectively.

In our main analysis, the rule-based model delivered greater precision than the CNN model (0.91 vs 0.71) for predicting the total EDSS score. Conversely, the CNN model had greater accuracy (0.86 vs 0.57) and slightly better recall (0.70 vs 0.65) relative to the rule-based model (Multimedia Appendix 1). In a qualitative error analysis of the validation set (n=3493 notes), the numbers and proportions of instances where the EDSS score was captured by both models, captured only by the rule-based method, captured only by the CNN, and missed by both models were 1864 (53.4%), 122 (3.5%), 1155 (33.1%), and 352 (10.1%), respectively. Model performance varied at the extremes of the EDSS score, with the rule-based approach performing worse at the lower ranges where patient disability is minimal, while the CNN model underpredicted EDSS scores in patients with very high levels of disability (Multimedia Appendix 2). Specifically, the F-scores for the rule-based and CNN models at EDSS scores of 0 to 4 were 0.69 and 0.89, respectively, while those for EDSS scores greater than 4 were 0.78 and 0.54, respectively. We observed similar patterns when comparing notes that did (n=2172, 62.2%) and did not (n=1321, 37.8%) report an EDSS score (Multimedia Appendix 3). For notes with an explicit EDSS score, the accuracies of the rule-based and CNN models were 0.87 and 0.93, respectively, with the rule-based model achieving greater performance at higher EDSS scores and slightly lower performance at lower EDSS scores, in part because of lower recall when the EDSS score is 0.0. For notes lacking an explicit EDSS score, the accuracy of the CNN model was 0.74, while the rule-based model was unable to return an EDSS score, with all predictions being labeled as “unknown.”

When compared with each model individually, the combined rule-based–CNN model performed best for predicting the total EDSS score, with accuracy, precision, recall, and an F-score of 0.90, 0.82, 0.83, and 0.83, respectively (Multimedia Appendix 1). We obtained similar results for the combined model using 10-fold cross-validation, with accuracy and an F-score of 0.87 and 0.81, respectively. The differences in accuracy and F1-score between the combined rule-based–CNN model and both the rule-based and CNN models were statistically significant (*P*<.001). The proportions of records with an unknown EDSS score prediction with the rule-based model, CNN model, and combined model were 44.43% (1552/3493), 3.06% (107/3493), and 2.83% (99/3493), respectively.

Similar to the total EDSS score, the combined model performed best for predicting EDSS functional system subscores (Multimedia Appendix 1). However, relative to the total EDSS score, functional system subscores had higher rates of unknown values in patient records, ranging from 8.2% for the ambulation subscore to 33.3% for the cerebral subscore. Consequently, performance measures were generally lower for combined models predicting EDSS functional system subscores relative to the total score (Multimedia Appendix 1). We therefore determined a post-hoc converted accuracy by excluding unknown values from the analysis and calculating performance metrics from notes with valid scores. The converted accuracy exceeded 0.90 for all EDSS functional system subscores, ranging from 0.94 for the sensory function subscore to 0.98 for brainstem and bowel/bladder function subscores.

## Discussion

In our study, we found that a combined rule-based–CNN natural language processing approach can accurately extract the EDSS score from the clinic notes of people with MS. Moreover, the combined model was able to derive the EDSS score in notes that did not explicitly contain this information using available MS-specific variables. These results highlight the feasibility of developing automated algorithms for the extraction of clinically relevant information that would be otherwise challenging to abstract manually from unstructured data sources.

Our work confirms and builds upon earlier work using natural language processing methods in the field of MS in several ways [9,17-24]. First, while previous studies have used rule-based approaches to develop classification algorithms for identifying patients with MS and extracting clinically relevant information from electronic health records, we compared 3 separate natural language processing models for extracting the EDSS score, demonstrating that the combination of a CNN and rule-based algorithm leverages the strengths of each method while overcoming the limitations inherent in each approach. Specifically, the rule-based model exhibited greater precision, excelling when the keyword “EDSS” and an associated score appeared explicitly in the note, but had lower recall, particularly for patients at the lowest extreme of EDSS scores where physicians may be more likely to provide a qualitative summary of a patient’s disability status with no accompanying EDSS score (eg, “neurological exam remains normal”). In such cases, the rule-based approach will return an EDSS score of “unknown,” signifying no extraction of any score. Additionally, the rule-based approach struggled with cases where there were multiple EDSS scores in the note (eg, “she previously had an EDSS score of 5.0 and her current score is of 6.0”), or when the EDSS score was written in a format not accounted for in our rules (eg, “EDSS was three”). These limitations were reduced by the CNN model, which derived an EDSS score using high-level text features in the note and performed well in predicting EDSS scores in the lower range. Conversely, class imbalance in the higher range of EDSS scores undermined the performance of the CNN model, resulting in underprediction of the EDSS score among the very few patients with extremely high scores (Multimedia Appendix 2). This weakness was mitigated when combined with the rule-based model, which performed well for high EDSS scores by capturing relevant keyword patterns. By combining the 2 models, we leveraged the strengths of each to optimize performance for both low and high EDSS scores.

Second, although previous studies have demonstrated that natural language processing models can extract the EDSS score and the related MS severity score from patient records containing these data [9,21,23], we demonstrated that a combined rule-based–CNN model could derive the EDSS score from notes where this measure was not explicitly provided, a phenomenon observed in approximately one-third of the notes available for study. The ability to automate EDSS score derivation using available clinical data may address issues of missing data within electronic health records and facilitate the use of these databases for quality improvement and research purposes.

Finally, we examined whether natural language processing models could extract functional EDSS subscores from electronic health records. Our model was able to extract the subscores, albeit with less precision than the total EDSS score. This is a line of inquiry that has not been addressed in prior studies.

Our study has some limitations. Although there were a sufficient number of notes available for ascertaining model performance related to the total EDSS score, data were sparser for our secondary analyses of the functional system subscores. These findings should therefore be considered hypothesis generating, and they warrant further evaluation with larger data sets. In addition, our models were developed and validated using the records of a single MS clinic embedded within a large academic teaching hospital. Consequently, the portability of our models is unknown. Finally, our models identify cross-sectional associations and cannot be considered as algorithms that predict disability progression in patients with MS. However, our models may automate the extraction of this information for use as inputs in future studies of machine learning approaches for predicting outcomes in patients with MS.

In conclusion, we found that a combined rule-based–CNN model was superior to either model alone for extracting and/or deriving EDSS scores from the records of patients with MS. This approach can be harnessed to establish and maintain clinical and research databases of people with MS, which may otherwise be too time-consuming and labor-intensive to maintain.

## Appendix

Multimedia Appendix 1 Model performance for predicting the total Expanded Disability Status Scale score and functional system subscores.

Multimedia Appendix 2 Perclass model performance for the rule-based, convolutional neural network, and combined models.

Multimedia Appendix 3 Performance of the rule-based, convolutional neural network, and combined models stratified by the presence or absence of the Expanded Disability Status Scale score in notes.

## Abbreviations

BERT: Bidirectional Encoder Representations from Transformers
CNN: convolutional neural network
EDSS: Expanded Disability Status Scale
MS: multiple sclerosis
RNN: recurrent neural network
