# Supplementary material for: Assessment of Natural Language Processing Methods for Ascertaining the Expanded Disability Status Scale Score From the Electronic Health Records of Patients With Multiple Sclerosis: Algorithm Development and Validation Study
Source: JMIR Med Inform. 2022 Jan 12;10(1):e25157. doi: 10.2196/25157 (PMC8792771; doi:10.2196/25157)
Supplement: Multimedia Appendix 1 [file medinform_v10i1e25157_app1.docx]

**Multimedia Appendix 1.** Model performance for predicting the total Expanded Disability Status Scale score and functional system subscores.

| **Variable** | **Model** | **Unknown rate (%)^a^** | **Predicted unknown rate (%)^b^** | **Accuracy** | **Macro** | | | **Converted accuracy** |
| --- | --- | --- | --- | --- | --- | --- | --- | --- |
|  |  |  |  |  | **Precision** | **Recall** | **F-score** |  |
| Total EDSS Score | Rule-based | 2.8 | 44.4 | 0.57 | 0.92 | 0.65 | 0.70 | N/A |
|  | CNN | 2.8 | 3.1 | 0.86 | 0.71 | 0.70 | 0.70 | N/A |
|  | Combined Model | 2.8 | 2.8 | 0.90 | 0.82 | 0.83 | 0.83 | N/A |
| Pyramidal Function | Rule-based | 9.8 | 23.5 | 0.53 | 0.50 | 0.47 | 0.44 | 0.7 |
|  | CNN | 9.8 | 7.0 | 0.75 | 0.62 | 0.55 | 0.57 | 0.98 |
|  | Combined Model | 9.8 | 4.9 | 0.65 | 0.55 | 0.52 | 0.50 | 0.97 |
| Cerebellar Function | Rule-based | 15.2 | 24.0 | 0.63 | 0.43 | 0.42 | 0.42 | 0.96 |
|  | CNN | 15.2 | 14.7 | 0.75 | 0.50 | 0.48 | 0.49 | 0.99 |
|  | Combined model | 15.2 | 10.3 | 0.70 | 0.47 | 0.45 | 0.45 | 0.97 |
| Brain Stem Function | Rule-based | 14.1 | 34.3 | 0.60 | 0.55 | 0.57 | 0.51 | 0.98 |
|  | CNN | 14.1 | 11.3 | 0.77 | 0.53 | 0.44 | 0.45 | 0.99 |
|  | Combined Model | 14.1 | 10.3 | 0.74 | 0.59 | 0.60 | 0.56 | 0.98 |
| Sensory function | Rule-based | 13.7 | 23.1 | 0.59 | 0.39 | 0.42 | 0.37 | 0.93 |
|  | CNN | 13.7 | 14.4 | 0.78 | 0.53 | 0.51 | 0.52 | 0.99 |
|  | Combined Model | 13.7 | 11.3 | 0.65 | 0.65 | 0.42 | 0.43 | 0.94 |
| Bladder and Bowel Function | Rule-based | 33.2 | 83.3 | 0.45 | 0.31 | 0.29 | 0.27 | 0.99 |
|  | CNN | 33.2 | 32.2 | 0.71 | 0.37 | 0.38 | 0.37 | 0.98 |
|  | Combined Model | 33.2 | 30.7 | 0.71 | 0.50 | 0.49 | 0.49 | 0.98 |
| Visual Function | Rule-based | 18.7 | 32.4 | 0.69 | 0.55 | 0.51 | 0.51 | 0.98 |
|  | CNN | 18.7 | 16.9 | 0.79 | 0.35 | 0.38 | 0.36 | 0.96 |
|  | Combined Model | 18.7 | 14.0 | 0.79 | 0.57 | 0.54 | 0.54 | 0.97 |
| Ambulation Function | Rule-based | 8.2 | 48.4 | 0.33 | 0.51 | 0.52 | 0.48 | 0.94 |
|  | CNN | 8.2 | 2.8 | 0.72 | 0.30 | 0.31 | 0.28 | 0.93 |
|  | Combined Model | 8.2 | 2.1 | 0.66 | 0.52 | 0.57 | 0.52 | 0.96 |
| Cerebral Function | Rule-based | 33.3 | 55.4 | 0.55 | 0.34 | 0.32 | 0.31 | 0.95 |
|  | CNN | 33.3 | 37.4 | 0.70 | 0.41 | 0.36 | 0.36 | 0.99 |
|  | Combined Model | 33.3 | 33.0 | 0.66 | 0.38 | 0.38 | 0.38 | 0.96 |

^a^ Proportion of extracted labels where EDSS score is missing

^b^ Proportion of labels for which model assigns a value of 'missing' to the label
