# Supplementary material for: Assessment of Natural Language Processing Methods for Ascertaining the Expanded Disability Status Scale Score From the Electronic Health Records of Patients With Multiple Sclerosis: Algorithm Development and Validation Study
Source: JMIR Med Inform. 2022 Jan 12;10(1):e25157. doi: 10.2196/25157 (PMC8792771; doi:10.2196/25157)
Supplement: Multimedia Appendix 2 [file medinform_v10i1e25157_app2.docx]

**Multimedia Appendix 2.** Perclass model performance for the rule-based, convolutional neural network, and combined models.

| **EDSS Class** | **N** | **CNN F-score** | **Rule-based F-score** | **Combined model F-score** |
| --- | --- | --- | --- | --- |
| **0** | 557 | 0.93 | 0.03 | 0.94 |
| **1** | 565 | 0.92 | 0.69 | 0.93 |
| **1.5** | 283 | 0.93 | 0.94 | 0.94 |
| **2** | 666 | 0.91 | 0.76 | 0.94 |
| **2.5** | 250 | 0.89 | 0.92 | 0.92 |
| **3** | 287 | 0.84 | 0.71 | 0.86 |
| **3.5** | 123 | 0.83 | 0.90 | 0.87 |
| **4** | 103 | 0.88 | 0.57 | 0.90 |
| **4.5** | 34 | 0.49 | 0.76 | 0.63 |
| **5** | 53 | 0.63 | 0.63 | 0.76 |
| **5.5** | 36 | 0.81 | 0.82 | 0.82 |
| **6** | 167 | 0.81 | 0.83 | 0.84 |
| **6.5** | 170 | 0.83 | 0.86 | 0.87 |
| **7** | 47 | 0.56 | 0.58 | 0.59 |
| **7.5** | 30 | 0.39 | 0.81 | 0.71 |
| **8** | 12 | 0.30 | 0.74 | 0.71 |
| **8.5** | 11 | 0.00 | 0.95 | 0.95 |
| **Unknown** | 99 | 0.65 | 0.12 | 0.67 |
