# Supplementary material for: Assessment of Natural Language Processing Methods for Ascertaining the Expanded Disability Status Scale Score From the Electronic Health Records of Patients With Multiple Sclerosis: Algorithm Development and Validation Study
Source: JMIR Med Inform. 2022 Jan 12;10(1):e25157. doi: 10.2196/25157 (PMC8792771; doi:10.2196/25157)
Supplement: Multimedia Appendix 3 [file medinform_v10i1e25157_app3.docx]

**Multimedia Appendix 3.** Performance of the rule-based, convolutional neural network, and combined models stratified by the presence or absence of the Expanded Disability Status Scale score in notes.

|  | **Notes with explicit EDSS score (n = 2172)** | | | | **Notes without an explicit EDSS score (n = 1321)** | | | |
| --- | --- | --- | --- | --- | --- | --- | --- | --- |
| **EDSS Score** | **N** | **Rule-based F-Score** | **CNN F-Score** | **Combined F-Score** | **N** | **Rule-based F-Score** | **CNN F-Score** | **Combined F-Score** |
| 0 | 128 | 0.13 | 0.97 | 0.97 | 429 | 0 | 0.92 | 0.92 |
| 1 | 330 | 0.95 | 0.98 | 0.98 | 235 | 0 | 0.83 | 0.83 |
| 1.5 | 256 | 0.99 | 0.98 | 0.99 | 27 | 0 | 0.13 | 0.13 |
| 2 | 463 | 0.94 | **0.96** | 0.99 | 203 | 0 | 0.8 | 0.8 |
| 2.5 | 223 | 0.98 | 0.95 | 0.98 | 27 | 0 | 0.13 | 0.13 |
| 3 | 191 | 0.9 | 0.95 | 0.96 | 96 | 0 | 0.64 | 0.64 |
| 3.5 | 105 | 0.98 | 0.94 | 0.98 | 18 | 0 | 0.07 | 0.07 |
| 4 | 49 | 0.9 | 0.93 | 0.93 | 54 | 0 | 0.83 | 0.83 |
| 4.5 | 25 | 0.9 | 0.73 | 0.88 | 9 | 0 | 0 | 0 |
| 5 | 33 | 0.85 | 0.83 | 0.92 | 20 | 0 | 0.2 | 0.21 |
| 5.5 | 30 | 0.9 | 0.9 | 0.90 | 6 | 0 | 0 | 0 |
| 6 | 130 | 0.95 | 0.94 | 0.97 | 37 | 0 | 0.44 | 0.44 |
| 6.5 | 136 | 0.97 | 0.92 | 0.97 | 34 | 0 | 0.49 | 0.49 |
| 7 | 27 | 0.83 | 0.86 | 0.90 | 20 | 0 | 0.26 | 0.26 |
| 7.5 | 24 | 0.92 | 0.45 | 0.92 | 6 | 0 | 0.26 | 0.26 |
| 8 | 7 | 1 | 0.3 | 0.93 | 5 | 0 | 0.31 | 0.31 |
| 8.5 | 10 | 1 | 0 | 1.00 | 1 | 0 | 0 | 0.00 |
| Unknown | 5 | 0 | 0.14 | 0.00 | 94 | 0.13 | 0.68 | 0.68 |
| Macro average |  | 0.84 | 0.76 | 0.90 |  | 0.01 | 0.39 | 0.39 |
